# Orally Formulated Artemisinin in Healthy Fasting Vietnamese Male Subjects: A Randomized, Four-Sequence, Open-Label, Pharmacokinetic Crossover Study

**DOI:** 10.1016/j.clinthera.2011.04.017

**Published:** 2011-05

**Authors:** Tran Tinh Hien, Warunee Hanpithakpong, Nguyen Thanh Truong, Nguyen Thi Dung, Pham Van Toi, Jeremy Farrar, Niklas Lindegardh, Joel Tarning, Michael Ashton

**Affiliations:** 1 Hospital for Tropical Diseases, Ho Chi Minh City, Vietnam; 2 Mahidol–Oxford Tropical Medicine Research Unit, Faculty of Tropical Medicine, Mahidol University, Bangkok, Thailand; 3 Oxford University Clinical Research Unit, South East Asia Infectious Disease Clinical Research Network, Hospital for Tropical Diseases, Ho Chi Minh City, Vietnam; 4 Centre for Clinical Vaccinology and Tropical Medicine, Churchill Hospital, Oxford, United Kingdom; 5 Department of Pharmacology, Sahlgrenska Academy, Göteborg University, Göteborg, Sweden

**Keywords:** ACT, artemisinin, bioequivalence, malaria, pharmacokinetics, piperaquine

## Abstract

**Background:**

Artemisinin derivatives are used in antimalarial drug combination therapy. Artemisinin and piperaquine have recently been proven to be prospective candidates for combination therapy in the treatment of uncomplicated *Plasmodium falciparum* malaria.

**Objective:**

The goal of this study was to evaluate the relative bioavailability and to characterize the pharmacokinetic properties of a new micronized powder formulation of artemisinin against the previous standard Vietnamese formulation when administered as a single oral dose or in combination with piperaquine.

**Methods:**

This was a single-center, randomized, 4-sequence, open-label, crossover study conducted in 15 healthy male Vietnamese volunteers under fasting conditions with a washout period of 3 weeks between study visits. A single oral dose of 160 or 500 mg of artemisinin was administered alone or in combination with piperaquine. Potential adverse events were monitored daily by the clinician and by using laboratory test results. Frequent blood samples were drawn for 12 hours after dose. Artemisinin was quantified in plasma using LC-MS/MS. Pharmacokinetic parameters were computed from the plasma concentration–time profiles using a noncompartmental analysis method.

**Results:**

Pharmacokinetic parameters T_max_, C_max_, AUC_0-∞_, V_d_/F, CL/F, and t_1/2_ (mean [SD]) for the new formulation of artemisinin were 1.83 (0.88) hours, 178 (97) ng/mL, 504 (210) h × ng/mL, 1270 (780) L, 401 (260) L/h, and 2.21 (0.29) hours, respectively. The mean percentage of the test/reference formulation ratio for the logarithmically transformed values of C_max_, AUC_0–last,_ and AUC_0–∞_ were 121% (90% CI, 92.5–158), 122% (90% CI, 101–148), and 120% (90% CI, 98.0–146), respectively.

**Conclusions:**

This single-dose study found that the dose-normalized C_max_, AUC_0–last_, and AUC_0–∞_ mean geometric differences between the test and reference formulations were relatively small (<40%) and will probably not have a clinical impact in the treatment of malaria infections.

## Introduction

Malaria remains a major health problem worldwide due to the emergence of multidrug-resistant *Plasmodium falciparum* parasite strains.^1^ In Vietnam and other parts of Southeast Asia, the parasites have developed resistance to almost all the drugs available on the market.^2^ Resistance to artemisinin derivatives has recently been reported on the Thai-Cambodian border, as indicated by increased parasite clearance times in patients with falciparum malaria.^3–8^ However, artemisinin derivatives (artemisinin, artesunate, artemether, arteether, and dihydroartemisinin) are still very effective, with <5% polymerase chain reaction (PCR)-confirmed parasitologic treatment failures at day 28 when treating multidrug-resistant falciparum strains with artemisinin-based combination therapy (ACT).^9–12^

Artemisinin monotherapy was registered in Vietnam as first-line treatment for a number of years in the 1990s. A major drawback with artemisinin monotherapy and its derivatives is the high recrudescence rate observed in clinical studies (∼10% recrudescence rate with 5 days of monotherapy).^13^ The short terminal t_1/2_ of the artemisinin derivatives (ie, 0.5–2 hours) has been suggested as the main reason for the resulting low efficacy when these drugs are used as monotherapy. The artemisinin derivatives have therefore been used in different combination treatments to reduce the high recrudescence rates observed with monotherapy and to prevent the development of parasite drug resistance by introducing another drug with a different mechanism of action.^14–16^ ACT is now recommended as first-line treatment worldwide for uncomplicated falciparum malaria.^17^

Artemisinin has not been used to a great extent in ACTs because of its time-dependent pharmacokinetics and relatively low bioavailability (<30%). Artemisinin and artemether, but not artesunate or dihydroartemisinin, reportedly have a marked ability for enzymatic autoinduction when administered continuously over several days.^18–23^ These time-dependent pharmacokinetics can be seen as an increase in the clearance rate after repeated oral administration. However, this pharmacokinetic characteristic should be less pronounced if artemisinin were used in a combination treatment that had a duration of therapy shorter than that used in monotherapy. The traditional oral artemisinin formulation has a low relative bioavailability of ∼30% compared with intramuscular oil suspension, and very low and erratic concentrations were reported after rectal administration in healthy volunteers.^24,25^ A new formulation has been developed containing smaller drug particles (ie, micronization) to increase the effective surface area after disintegration and deaggregation. This change is believed to improve dissolution of oral artemisinin and therefore also the relative bioavailability.^26^

Piperaquine has been used for decades as monotherapy and was introduced as a partner drug in combination therapy with artemisinin derivatives during the 1990s in China.^27^ The fixed oral combination of piperaquine and dihydroartemisinin is reportedly well tolerated (early vomiting, 1.7%) with high efficacy (PCR-corrected cure rate of 98.7% at day 28) in adults with uncomplicated malaria.^12^ This fixed combination reportedly has a slightly lower efficacy in children (PCR-corrected cure rate of 94.2% at day 28). Tolerability and efficacy of a new fixed oral combination of artemisinin-piperaquine and the commonly used dihydroartemisinin-piperaquine combination have recently been reported in 103 Vietnamese adult patients with uncomplicated falciparum malaria.^28^ In patients receiving the artemisinin-piperaquine combination, there were no significant differences (mean [SD]) in parasite clearance times (43.2 [13.9] vs 36.5 [17.1] hours) or in fever clearance times (24.2 [9.9] vs 22.7 [11.2] hours) when compared with the commonly used dihydroartemisinin-piperaquine combination. Both combinations resulted in a 100% cure rate with no recrudescent malaria at day 28, but the artemisinin-piperaquine combination resulted in a lower incidence of gastrointestinal adverse events than the dihydroartemisinin-piperaquine combination. The artemisinin-piperaquine combination has a simplified dosing scheme (2-day therapy) compared with commonly used ACTs (3- to 6-day therapy), which may increase patient compliance and consequently the efficacy of the treatment. However, a shorter treatment regimen might not be sufficient in some areas and should be used with caution because it may cause artemisinin resistance to emerge more quickly. To the best of our knowledge, no pharmacokinetic information of this novel artemisinin-piperaquine combination can be found in the international published literature.

The main objective of the present study was to determine if the micronization of artemisinin powder would increase its oral bioavailability. This hypothesis was explored by investigating the pharmacokinetics of artemisinin in a micronized formulation (micronized artemisinin powder encapsulated and in a fixed oral combination with piperaquine in tablet formulation⁎) compared with the reference formulation of previously used standard Vietnamese formulation (encapsulated artemisinin powder) when administered as a single oral dose. A secondary objective was to investigate the potential influence of piperaquine on artemisinin pharmacokinetics.

## Subjects and Methods

### Study Subjects

Fifteen healthy Vietnamese male volunteers aged 18 to 55 years were enrolled in the study. No female subjects were enrolled to avoid potential risks of adverse effects on the fetus during early pregnancy. The study design and potential adverse drug effects were explained to all volunteers in their own language before initiation of any study-related procedures. Volunteers who provided written informed consent were considered for enrollment. Clinical and laboratory screenings were performed at the Hospital for Tropical Diseases in Ho Chi Minh City (HTD-HCMC), Vietnam, and the results evaluated before enrollment. Clinical evaluation and laboratory assessment (full hematology and biochemistry) were also performed at follow-up 1 week after the last study visit.

A volunteer was ineligible to participate in the study if any of the following criteria was met: intake of any antimalarial agent during the previous 3 months, participation in an ongoing clinical drug study or within the last 3 months, involvement in the planning and/or conduct of the study, inability to comply with study procedure during the 10 weeks of participation, or intending to donate blood within 6 months after study start. Healthy volunteers, according to clinical and laboratory screening data, who fulfilled all of the inclusion but none of the exclusion criteria were enrolled in the study.

### Study Drugs and Design

This was a single-center, single-dose, open-label, randomized, 4-sequence, crossover study with a 3-week washout period (ie, >5 artemisinin half-lives) between occasions. The study was conducted at HTD-HCMC. The clinical trial protocol was reviewed and supported by the internal Scientific and Ethical Committee of the HTD-HCMC and Oxford Tropical Research Ethics Committee (OxTREC 019-06), University of Oxford, Oxford, United Kingdom. The volunteers were randomly assigned to 1 of the 24 possible sequences for the 4 treatment groups (Table I). Group T1 received a single dose of 2 hard gelatin capsules⁎ (size 2), each containing 80 mg of micronized artemisinin powder (test formulation); group T2 received a single dose of 2 hard gelatin capsules, each containing 80 mg of artemisinin powder (reference Vietnamese low-dose formulation); group T3 received a single dose of 2 hard gelatin capsules, each containing 250 mg of artemisinin powder (reference Vietnamese dose-strength formulation); and group T4 received a single dose of 2 tablets, each containing 80 mg of micronized artemisinin (test formulation) and 360 mg of piperaquine phosphate. A fixed tablet combination of piperaquine and the reference Vietnamese formulation is not commercially available and could not be included in this trial.

Micronized artemisinin powder for group T1 was produced by Guangzhou University of Traditional Chinese Medicine, Guangzhou, China (batch number: 20030701) and encapsulated by STADA pharmaceutical joint-venture company, Ho Chi Minh City, Vietnam (batch number: NC01-20050920). Artemisinin powder for groups T2 and T3 was produced by Mediplantex, Hanoi, Vietnam (batch number: VN 97) and encapsulated by STADA (batch numbers: NC02-20050920 and NC03-20050920). The tablets for group T4 were produced by Artepharm Co., Ltd., Guangzhou, China (batch number: 20050301). The artemisinin content of filled capsules was analyzed by the Unit for Pharmacokinetics and Drug Metabolism, Department of Pharmacology, University of Gothenburg, Sweden, using a HPLC method with ultraviolet detection.^29^ Capsules were found to contain a mean (SD) artemisinin content of 85.8 (1.5) mg, 86.7 (0.75) mg, and 254 (2.6) mg in 9 capsules of groups T1, T2, and T3, respectively.

Study drug was administered with 200 mL of drinking water after an overnight fast of >10 hours, according to US Food and Drug Administration (FDA) guidelines for bioequivalence studies.^30^ The volunteers were asked to continue fasting for 2 hours after drug administration. Beverages containing caffeine or alcohol were not allowed on days of drug administration and blood sampling. Concomitant food intake was previously reported to have no effect on artemisinin pharmacokinetics^31^ and thus not studied further here. Tolerability was assessed daily by the clinician during each study occasion (using an adverse-event report form) and at the follow-up visit 1 week after last study occasion (using an adverse-event report form and laboratory assessment). Adverse events were accessed by using an open question about potential health problems during the study and followed up with a questionnaire if any health problems had occurred since the last consultation.

### Blood Sampling

An intravenous indwelling cannula was introduced and maintained in the antecubital vein and kept for 12 hours during blood sample collection at each study visit. A blood volume of 0.5 mL was discarded before sample collection to avoid drug dilution effects. A5-mL blood sample was drawn into lithium heparin tubes (Hong Thien My Medical Equipment Joint Stock Co., Ho Chi Minh City, Vietnam) before drug administration (predose) and at the following times after drug administration (postdose): 0.25, 0.5, 1, 1.5, 2, 2.5, 3, 4, 5, 6, 7, 8, 10, and 12 hours. The same time schedule was followed for all study visits. Saline solution (0.9% sodium chloride, 2 mL) was used to flush the lines after blood collection. Blood samples were immediately centrifuged at 3000*g* for 10 minutes at 20°C. Plasma were transferred to cryotubes within 10 minutes after centrifugation and stored directly at –70°C. All samples were freighted to Thailand on dry ice and analyzed by the Clinical Pharmacology Laboratory at Mahidol–Oxford Tropical Medicine Research Unit (Bangkok, Thailand).

### Drug Analysis

Plasma concentrations of artemisinin were determined using LC-MS/MS.^32^ The LC-MS/MS system consisted of an Agilent 1200 LC system including an autosampler set at 4°C and a column oven set at 40°C (Agilent Technologies Inc., Santa Clara, California) coupled with an API 5000 triple quadrupole mass spectrometer (Applied Biosystems/MDS SCIEX, Foster City, California). The lower limit of quantitation was set to 1.03 ng/mL, with a limit of detection of 0.257 ng/mL. Briefly, 50 μL of plasma was mixed with internal standard (ie, artesunate), and the samples were extracted on a 96-well μElution HLB solid-phase extraction plate (Waters Corporation, Milford, Massachusetts). Three independent quality-control samples in plasma (2.89, 40.7, and 571 ng/mL) were prepared fresh and analyzed in triplicate in each batch of extracted plasma samples. All samples were kept on ice until loaded onto the solid-phase extraction plate. The extracted samples were injected directly (5 μL) into the LC-MS/MS system.

### Pharmacokinetic and Statistical Analysis

Individual artemisinin plasma concentration–time data were evaluated using a noncompartmental analysis approach as implemented in WinNonlin version 5.0 (Pharsight Corporation, Moutainview, California). AUC_0–last_ values were calculated using the linear trapezoidal method for ascending concentrations and the logarithmic trapezoidal method for descending concentrations.^33^ Artemisinin exposure was extrapolated from AUC_0–∞_ by C_last_/λ_z_ for each individual subject to compute total drug exposure. The terminal t_1/2_ was estimated by log-linear regression of 5 to 7 observed concentrations. C_max_ and T_max_ concentrations were taken directly from the observed data. V_d_/F and CL/F were computed individually according to standard procedures and summarized for each treatment group. A difference of <50% was assumed to not be clinically significant, and the study was powered to detect a minimum difference of 40% in pharmacokinetic parameters (β = 0.8, α = 0.05, %CV [intraindividual] = 0.3).

Descriptive statistics and ANOVA with a 95% CI for the regression coefficient were conducted on individual logarithmically transformed pharmacokinetic parameters (ie, AUC_0–last_, AUC_0–last_/D, AUC_0–∞_, AUC_0–∞_/D, C_max_, C_max_/D, T_max_, CL/F, V_d_/F, and t_1/2_), were *D* equaled dose, using Stata version 10 (Stata Corporation, College Station, Texas). The model allowed for examining the effects of formulation, sequence, and subjects. Average bioequivalence was evaluated according to the FDA's guidelines for bioequivalence.^30,34^ The criteria for assuming bioequivalence is met if the 90% CI values for geometric mean ratios of AUC_0–∞_, AUC_0–last_, and C_max_ for the test formulation over the reference formulation fall within 80% to 125%. The point estimate of the geometric mean ratio and the residual variability from the ANOVA were used to calculate the 90% CIs around the mean to assess bioequivalence. Geometric mean ratios and CIs for dose-normalized (milligram per kilogram) parameters were also calculated to compensate for different body weights in the studied population.

## Results

### Artemisinin Pharmacokinetics

Fifteen Vietnamese male volunteers were enrolled and completed the study. Full demographic characteristics are given in Table II.

**Figure 1:**
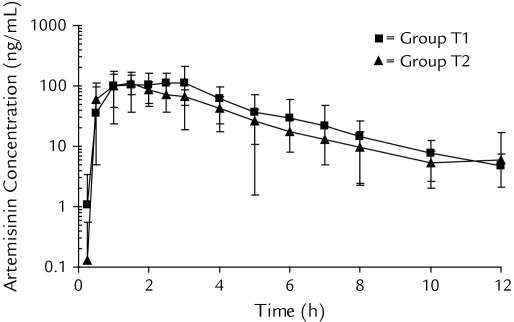
Mean (SD) plasma concentration–time curve of artemisinin in 15 healthy Vietnamese male volunteers in a 4-sequence crossover study receiving a single oral dose of 160-mg micronized test artemisinin formulation (group T1 ■) and 160-mg reference artemisinin formulation (group T2 ▲). Artemisinin concentrations are plotted on a log-linear scale with the base 10.

**Figure 2:**
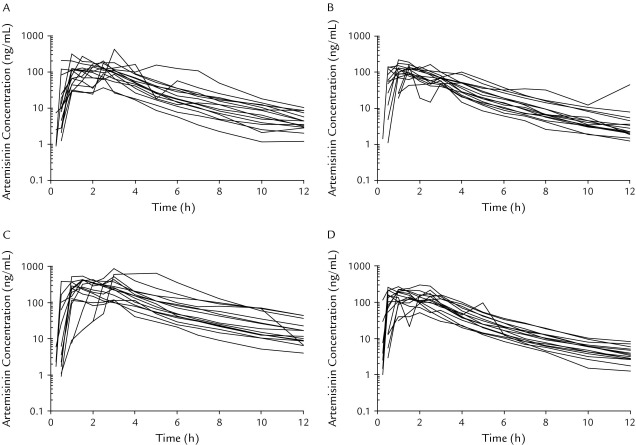
Individual plasma concentration–time curves of artemisinin in 15 healthy Vietnamese male volunteers in a 4-sequence crossover study. (A) Group T1 received a single dose of 2 gelatin capsules, each containing 80 mg of micronized artemisinin powder (test formulation); (B) Group T2 received a single dose of 2 gelatin capsules, each containing 80 mg of artemisinin (reference Vietnamese low-dose formulation); (C) Group T3 received a single dose of 2 gelatin capsules, each containing 250 mg of artemisinin powder (reference Vietnamese dose-strength formulation); and (D) Group T4 received a single dose of 2 tablets of micronized artemisinin powder, each containing 80 mg artemisinin (test formulation) and 360 mg of piperaquine phosphate. Artemisinin concentrations are plotted on a log-linear scale with the base 10.

The concentration–time profiles of oral artemisinin after different dosages and formulations were well captured by the applied sampling schedule in these healthy volunteers (Figures 1 and 2). Individual pharmacokinetic parameters in the different treatment periods were computed and are summarized in Table III. Mean (SD) percentages of artemisinin AUC_0–∞_ extrapolated from the last observed concentration to infinity were 2.94% (0.70%), 4.37% (7.4%), 4.29% (2.3%), and 2.43% (0.74%) for groups T1, T2, T3 and T4, respectively. All formulations were rapidly absorbed from the gastrointestinal tract, with measurable plasma artemisinin concentrations within 15 minutes to 1.5 hours after drug administration (Figure 2). Pooled data (n = 60) from all treatments show that artemisinin plasma concentrations peaked at a mean of 1.64 (0.92) hours after dosing and could be quantified in all patients for up to 12 hours. Artemisinin was rapidly eliminated, with a high mean CL/F of 420 (220) L/h, a short mean t_1/2_ of 2.23 (0.44) hours, and a large mean V_d_ of 1320 (620) L. There was also a large interindividual variability in artemisinin pharmacokinetic parameters, with a %CV between 13% and 65%.

As expected, statistically (*P* < 0.001) higher values for C_max_, AUC_0–last_, and AUC_0–∞_ were seen in the group receiving a higher dose (group T3, 500 mg of artemisinin) compared with the other groups (groups T1, T2, and T4, 160 mg of artemisinin). However, differences in milligram per kilogram dose-normalized AUC and C_max_ values did not reach statistical significance (*P* > 0.05) when artemisinin was administered as a 500-mg dose (group T3) compared with a 160-mg dose (group T2).

The micronized test formulation (group T1) resulted in 25% higher mean AUC and C_max_ values compared with the reference formulation (group T2), but it did not reach statistical significance (*P* = 0.151, *P* = 0.052, and *P* = 0.086 for C_max_, AUC_0–last_, and AUC_0–∞_, respectively) due to high intraindividual variability. No statistically significant differences were seen in other pharmacokinetic parameters between different treatment groups receiving artemisinin alone (groups T1, T2, and T3).

The fixed artemisinin-piperaquine tablet formulation (group T4) resulted in a 35% higher mean AUC and C_max_ values compared with the reference formulation (group T2) (*P* = 0.043, *P* = 0.009, and *P* = 0.018 for C_max_, AUC_0–last_, and AUC_0–∞_, respectively). However, no statistically significant differences in any pharmacokinetic parameters could be seen when micronized artemisinin powder was administered as a co-formulated fixed oral treatment with piperaquine (group T4) compared with when administered alone (group T1).

Bioequivalence could not be assumed because the 90% CIs for geometric mean ratios of dose-normalized and non-normalized AUC_0–∞_, AUC_0–last_, and C_max_ extended beyond the stipulated 80% to 125% limit according to the FDA guidelines (Table IV). ANOVA analysis revealed no significant (*P* > 0.18) sequence effects for the evaluated pharmacokinetic parameters.

### Tolerability

All of the volunteers completed the study and no adverse events were reported by any of the study subjects. No subject vomited after drug administration. There was a small but significant difference (*P* < 0.05) between enrollment and at follow-up in measured urea, glucose, creatinine clearance, bilirubin, protein, globulin, albumin/globulin, magnesium, aspartate aminotransferase level, and leukocyte, basophil, and lymphocyte counts in some subjects (Table V). The different values at enrollment and follow-up were in the normal range and therefore considered reversible, minor, or of no clinical relevance by the study physician. One subject had abnormally high alanine aminotransferase levels (120 U/L) at the follow-up visit 1 week after the last dose occasion. This increase was reversible, and the alanine aminotransferase level returned to normal 1 week later (ie, 2 weeks after the last dose). All subjects had normal ECG results at baseline and at the end of study (follow-up).

## Discussion

Pharmacokinetic profiles of artemisinin revealed higher drug concentrations after administration of micronized artemisinin (groups T1 and T4) compared with the reference formulation (group T2). The higher drug exposure suggests an increased bioavailability by micronization, but the dose-normalized C_max_, AUC_0–last_, and AUC_0–∞_ mean geometric differences between micronized artemisinin formulations and the reference formulation were relatively small (<40%) and might not be of clinical significance in the treatment of malaria infections. However, these results are derived from a single-dose study in healthy Vietnamese volunteers and should not be directly extrapolated to a population with malaria. No statistically significant differences in pharmacokinetic parameters could be seen when micronized artemisinin was administered alone (group T1) compared with when it was administered in combination with piperaquine (group T4). This finding suggests that piperaquine should not influence the pharmacokinetic characteristics of artemisinin when co-administered in the proposed fixed oral combination. The new micronized formulations did not meet the FDA criteria for assuming bioequivalence with the standard Vietnamese powder formulation. No sequence effect could be seen in this study, which suggests that any potential autoinduction of artemisinin metabolic capacity had normalized within 3 weeks after a single dose.

The pharmacokinetics of artemisinin seen in this study (mean values of CL/F, 7.26 L/h/kg; V_d_/F, 22.8 L/kg; and t_1/2_, 2.23 hours) were in good agreement with those reported previously in healthy Vietnamese male volunteers after a single oral administration of artemisinin (mean values: CL/F, 7.64, 5.10, and 5.38 L/h/kg, respectively; V_d_/F, 35.9, 19.4, and 16.4 L/kg; and t_1/2_, 2.10, 2.59, and 2.51 hours).^31,35,36^ Artemisinin pharmacokinetics were comparable to that reported in Vietnamese patients with liver cirrhosis (Child-Pugh class B^37^), indicating no altered clearance in patients with an underlying liver disease.^38^ Artemisinin has previously been reported to display significant dose-dependent pharmacokinetics in healthy volunteers after administration of 3 different doses (250, 500, and 1000 mg).^36^ Those healthy volunteers reported an increase in the terminal elimination t_1/2_ and a decrease in oral clearance with increasing doses, resulting in a nonlinear increase in drug exposure when normalized for different dose amounts. A similar dose dependency was observed in patients with malaria.^39^ This phenomenon was suggested to be a result of saturated first-pass extraction. Dose-dependent pharmacokinetics were not seen in the present study, which may be a consequence of the narrow range of doses studied here (160 and 500 mg).

Pooled pharmacokinetic data of smokers and nonsmokers from all treatment arms were analyzed to evaluate the effect of nicotine on pharmacokinetics. Smokers had a significantly higher (*P* = 0.024) oral clearance, with a subsequent lower milligram per kilogram dose-normalized drug exposure compared with nonsmokers. To the best of our knowledge, only 1 previous study has provided information about the effect of nicotine on artemisinin pharmacokinetics in healthy volunteers.^40^ That study reported similar pharmacokinetic parameters for the 2 groups. Artemisinin in vitro metabolism is mediated primarily by cytochrome P450 (CYP) 2B6, with a minor contribution from CYP2A6 and CYP3A4.^41^ In vitro studies have reported that CYP2A6 is the most important CYP enzyme in the metabolism of nicotine.^42,43^ Consequently, smoking should not have any effect on artemisinin clearance or result in a slightly slower clearance due to competitive inhibition of drug-metabolizing enzymes. However, there were only 3 nonsmokers of the 15 volunteers in this study, and the nicotine effect on artemisinin pharmacokinetics therefore must be confirmed in larger studies.

Artemisinin is generally a drug with good tolerability at therapeutic doses; no adverse events were reported by the subjects in this study. Biochemistry analysis showed small variations over time and a significant difference in a few patients at follow-up compared with baseline. Six of the 15 volunteers had higher levels of aspartate aminotransferase at follow-up compared with baseline levels. These higher levels were still in the normal range and of no clinical significance. No changes were seen in alanine aminotransferase levels except in 1 subject who had high levels at follow-up; these high levels returned to normal range within another week. Previous studies have reported a significant but transient increase in liver enzymes for artemisinin and dihydroartemisinin.^20,44^ Values for these patients returned to baseline ∼2 weeks after the study. The follow-up visit in this study was only 1 week after treatment, which may explain why the biochemistry values in some subjects were still elevated.

## Conclusions

This single-dose study of artemisinin found that the micronized test formulations resulted in a higher drug exposure compared with the reference formulation, but this difference was relatively small and will probably not have a clinical impact in the treatment of malaria infections. All formulations were well tolerated, and pharmacokinetics were comparable to what has been reported previously. Further studies are needed to investigate the pharmacokinetic properties of this formulation in patients with malaria.

## Figures and Tables

**Table I:** Sequences of administered study treatment (T) for each subject.

Visit	Subject														
1	2	3	4	5	6	7	8	9	10	11	12	13	14	15
1	T1	T1	T1	T2	T2	T2	T2	T3	T3	T3	T3	T4	T4	T4	T4
2	T2	T3	T4	T1	T1	T3	T4	T1	T1	T2	T2	T2	T2	T3	T3
3	T3	T4	T3	T3	T4	T1	T3	T2	T4	T1	T4	T1	T3	T1	T2
4	T4	T2	T2	T4	T3	T4	T1	T4	T2	T4	T1	T3	T1	T2	T1

**Table II:** Demographic data at enrollment for the 15 healthy Vietnamese male volunteers in this 4-sequence crossover study of artemisinin.⁎

Parameter	Value, Mean (SD) [Range]
Age, y	28.1 (8.5) [19–41]
Weight, kg	59.0 (9.3) [43–80]
Height, cm	166 (6.4) [158–180]
Systolic blood pressure, mm Hg	115 (7.4) [100–130]
Diastolic blood pressure, mm Hg	64.0 (6.3) [60–80]
Heart rate, beats/min	80.3 (4.2) [70–86]

⁎ Twelve of the 15 volunteers (80%) were smokers.

**Table III:** Noncompartmental analysis of artemisinin (ART) in 15 healthy Vietnamese male volunteers after a single oral dose. All data as mean (SD) [range].

Parameters	T1/New Formulation (160 mg ART)	T2/Standard Formulation (160 mg ART)	T3/Standard Formulation (500 mg ART)	T4/Fixed Tablets (160 mg ART + 720 mg PQ)
C_max_, ng/mL	178 (97) [36.4–421]	132 (35) [79.6–216]	403 (190)⁎ [120–855]	182 (68)⁎ [51.1–303]
C_max_/D, kg × ng/mL × mg	64.8 (37) [13.0–171]	49.3 (19) [26.9–108]	46.4 (21) [13.7–92.3]	67.1 (26)⁎ [18.2–102]
T_max_, h	1.83 (0.88) [0.500–3.00]	1.43 (0.84) [0.500–4.00]	1.94 (1.1) [0.567–5.00]	1.37 (0.72) [0.500–2.50]
AUC_0–last_, ng/mL/h	489 (200) [126–816]	380 (120) [192–661]	1300 (670)⁎ [422–2630]	510 (180)⁎ [170–908]
AUC_0–last_/D, kg × h × ng/mL × mg	177 (68) [44.8–272]	140 (48) [68.6–240]	148 (67) [48.1–263]	186 (63.5)⁎ [60.4–306]
AUC_0–∞_, ng/mL/h	504 (210) [129–838]	405 (150) [197–734]	1360 (710)⁎ [436–2730]	523 (190)⁎ [173–935]
AUC_0–∞/D_, kg × h × ng/mL × mg	182 (71) [46.1–280]	149 (62) [70.1–298]	155 (71) [49.7–273]	190 (66)⁎ [61.5–315]
t_1/2_, h	2.21 (0.29) [1.54–2.65]	2.27 (0.45) [1.76–3.49]	2.29 (0.43) [1.52–3.21]	2.24 (0.18) [1.80–2.50]
CL/F, L/h	401 (260) [191–1240]	445 (160) [218–813]	479 (270) [183–1148]	357 (180)⁎ [171–926]
V_d_/F, L	1270 (780) [423–3620]	1420 (490) [910–2430]	1560 (860) [454–3800]	1130 (450)⁎ [554–2420]

T1 = micronized artemisinin powder (160 mg); T2 = standard Vietnamese artemisinin formulation (160 mg); T3 = standard Vietnamese artemisinin formulation (500 mg); T4 = micronized artemisinin powder (160 mg) and piperaquine phosphate (720 mg) in a fixed tablet formulation; PQ = piperaquine phosphate; D = dose (milligram per kilogram).

⁎ Significant difference at *P* < 0.05 compared with reference treatment (group T2) using between-group ANOVA.

**Table IV:** Bioequivalence of artemisinin in 15 healthy Vietnamese male volunteers in a 4-sequence crossover study. Values are given as geometric mean values (90% CI).

Parameter	T1/T2 Ratio	T3/T2 Ratio	T4/T2 Ratio
C_max_	121% (92.5–158)	278% (213–363)	131% (100–171)
C_max_/D	121% (92.5–158)	89.0% (68.2–116)	131% (100–171)
AUC_0–last_	122% (101–148)	315% (260–381)	131% (109–159)
AUC_0–last_/D	122% (101–148)	101% (83.2–122)	131% (109–159)
AUC_0–∞_	120% (98.0–146)	313% (256–383)	128% (105–157)
AUC_0–∞_/D	120% (98.0–146)	100% (82.0–123)	128% (105–157)

T1 = micronized artemisinin powder (160 mg); T2 = standard Vietnamese artemisinin formulation (160 mg); T3 = standard Vietnamese artemisinin formulation (500 mg); T4 = micronized artemisinin powder (160 mg) and piperaquine phosphate (720 mg) in a fixed tablet formulation; D = dose (milligram per kilogram).

**Table V:** Baseline and follow-up laboratory data for the 15 healthy Vietnamese male volunteers in this 4-sequence crossover artemisinin study. All parameters are presented as mean values (SD) [range].

Variable	Pretreatment	Posttreatment	*P*
Biochemistry			
Urea, mM	3.49 (0.91) [2.4–5.2)	5.36 (1.5) [2.4–7.2]	0.001⁎
Glucose, mM	4.46 (0.68) [3.5–6.5]	3.91 (0.35) [3.5–4.4]	0.002⁎
Creatinine, μM	72.5 (8.9) [54–92]	92.7 (16) [73–115]	0.001⁎
Total bilirubin, μM	9.53 (2.7) [5.0–13]	16.1 (4.0) [9.0–20]	0.001⁎
Total protein, g/L	71.3 (2.1) [68–74]	77.3 (5.1) [67–82]	0.001⁎
Albumin, g/L	39.7 (1.8) [36–42]	39.7 (4.2) [30–46]	0.930
Globulin, g/L	31.5 (2.3) [28–36]	37.7 (6.5) [30–47]	0.002⁎
Albumin/globulin ratio	1.25 (0.14) [1.0–1.5]	1.08 (0.19) [0.80–1.4]	0.003⁎
Ferritin, μg/L	126 (57) [55–280]	117 (36) [69–190]	0.520
Magnesium, mM	1.09 (0.20) [0.80–1.5]	0.896 (0.17) [0.70–1.1]	0.001⁎
AST, U/L	20.5 (8.70 [11–36]	29.1 (16) [19–80]	0.001⁎
ALT, U/L	35.3 (18) [11–68]	44.7 (25) [15–120]	0.190
Hematology			
Hematocrit, %	46.8 (2.8) [43–54]	47.7 (2.5) [45–53]	0.680
Hemoglobin, g/dL	15.6 (0.85) [14–18]	15.6 (0.78) [15–18]	0.770
Erythrocytes, 10^12^/L	5.33 (0.51) [4.8–6.1]	5.23 (0.49) [4.6–6.1]	0.100
Reticulocytes, %	0.653 (0.58) [0.10–2.5]	0.700 (0.34) [0.10–1.5]	0.520
Thrombocytes, 10^9^/L	252 (51) [150–320]	258 (59) [130–330]	0.360
Leukocytes, 10^9^/L	8.49 (1.7) [5.9–12]	7.62 (1.5) [5.4–10]	0.015⁎
Neutrophils, %	56.4 (7.1) [47–70]	53.7 (6.5) [45–63]	0.120
Eosinophils, %	5.15 (5.3) [0.87–21]	5.34 (3.2) [1.6–13]	0.110
Basophils, %	0.808 (0.33) [0.24–1.4]	1.12 (0.49) [0.23–2.1]	0.018⁎
Monocytes, %	6.96 (1.4) [5.2–11]	7.47 (1.4) [5.3–10]	0.210
Lymphocytes, %	30.6 (6.3) [19–38]	32.5 (6.2) [21–43]	0.041⁎

AST = aspartate aminotransferase; ALT = alanine aminotransferase.

⁎ Significant difference at *P* < 0.05 between pretreatment and posttreatment values using a Wilcoxon signed rank test.

## Footnotes

⁎ Trademark: Artequick^®^ (Artepharm Co., Ltd., Guangzhou, China).

⁎ Trademark: Coni-Snap^®^ Capsules (Capsugel [Thailand] Co., Ltd., Ayutthaya, Thailand).
